# Identification and characterisation of the angiotensin converting enzyme-3 (ACE3) gene: a novel mammalian homologue of ACE

**DOI:** 10.1186/1471-2164-8-194

**Published:** 2007-06-27

**Authors:** Monika Rella, Joann L Elliot, Timothy J Revett, Jerry Lanfear, Anne Phelan, Richard M Jackson, Anthony J Turner, Nigel M Hooper

**Affiliations:** 1 Institute of Molecular and Cellular Biology, Faculty of Biological Sciences, University of Leeds, Leeds, LS2 9JT, UK; 2 Leeds Institute of Genetics, Health and Therapeutics, University of Leeds, Leeds, LS2 9JT, UK; 3 Pfizer Ltd, Sandwich, Kent, CT13 9NJ, UK

## Abstract

**Background:**

Mammalian angiotensin converting enzyme (ACE) plays a key role in blood pressure regulation. Although multiple ACE-like proteins exist in non-mammalian organisms, to date only one other ACE homologue, ACE2, has been identified in mammals.

**Results:**

Here we report the identification and characterisation of the gene encoding a third homologue of ACE, termed ACE3, in several mammalian genomes. The ACE3 gene is located on the same chromosome downstream of the ACE gene. Multiple sequence alignment and molecular modelling have been employed to characterise the predicted ACE3 protein. In mouse, rat, cow and dog, the predicted protein has mutations in some of the critical residues involved in catalysis, including the catalytic Glu in the HEXXH zinc binding motif which is Gln, and ESTs or reverse-transcription PCR indicate that the gene is expressed. In humans, the predicted ACE3 protein has an intact HEXXH motif, but there are other deletions and insertions in the gene and no ESTs have been identified.

**Conclusion:**

In the genomes of several mammalian species there is a gene that encodes a novel, single domain ACE-like protein, ACE3. In mouse, rat, cow and dog ACE3, the catalytic Glu is replaced by Gln in the putative zinc binding motif, indicating that in these species ACE3 would lack catalytic activity as a zinc metalloprotease. In humans, no evidence was found that the ACE3 gene is expressed and the presence of deletions and insertions in the sequence indicate that ACE3 is a pseudogene.

## Background

Angiotensin-converting enzyme (ACE; EC 3.4.15.1) is a well-characterised zinc metallopeptidase that plays a key role in the renin-angiotensin system [1]. Through cleavage of a C-terminal dipeptide from angiotensin I to produce the potent vasoconstrictor angiotensin II, and through inactivation of the vasodilator bradykinin, ACE regulates blood pressure and cardiovascular homeostasis. Inhibitors of ACE, such as captopril and lisinopril, are front line therapeutics in a range of cardiovascular disorders, including hypertension, congestive heart failure, left ventricular hypertrophy and myocardial infarction [2].

ACE exists in two forms as a result of alternative use of promoters within the same gene on human chromosome 17 and mouse chromosome 11: the larger, two domain somatic ACE (1306 amino acids in humans) and the smaller, single domain germinal ACE (701 amino acids in humans) [3]. The latter is identical to the C-terminal domain of somatic ACE, except for a unique region of 67 amino acids at its N-terminus. The two domains of somatic ACE are catalytically active and each contains the prototypical zinc binding motif HEXXH. In this motif, the two His residues are two of the zinc ligands, the third being a Glu on the C-terminal side of this motif [4]. The Glu in the HEXXH motif is critically involved in catalysis, binding the activated water molecule which initiates a nucleophilic attack on the susceptible peptide bond in the substrate. Recently, the separate three-dimensional structures of the C-domain and of the N-domain of ACE in complex with the inhibitor lisinopril have been reported [5,6]. Somatic and germinal ACE are synthesised with an N-terminal signal sequence that is cleaved off in the lumen of the endoplasmic reticulum and exist as type I integral membrane proteins anchored to the plasma membrane through a hydrophobic transmembrane domain at the C-terminus.

In 2000, we and another group independently reported the identification using genomics-based strategies of the first human homologue of ACE, termed angiotensin-converting enzyme-2 (ACE2) [7,8]. Like ACE, ACE2 is a type 1 integral membrane protein, however, ACE2 contains only a single active site domain and consists of 805 amino acids. The gene encoding ACE2 is located on the X chromosome. ACE2 acts as a carboxypeptidase removing single amino acids from the C-terminus of its substrates, whereas ACE acts predominantly as a peptidyl dipeptidase removing C-terminal dipeptides [1]. Studies from knockout mice indicate that ACE2 is an essential regulator of heart function [9] and is involved in the tissue response to injury [10]. ACE2 is also the receptor for the severe acute respiratory syndrome (SARS) coronavirus [11]. Homology modelling of the active site of ACE2 on the crystal structure of the C-domain of ACE [12] and the subsequent elucidation of the three-dimensional structure of the extracellular domain of ACE2 [13] revealed that the catalytic mechanism of ACE2 closely resembles that of ACE. However, the substrate-binding pockets differ significantly explaining the differences in substrate specificity between the two enzymes and the failure of ACE inhibitors to bind to and inhibit ACE2 [7,14].

ACE-like proteins also occur in non-mammalian species [15,16]. *Drosophila melanogaster *has two functionally active, single domain, soluble ACE-like proteins (termed Ance and Acer) that share 36% amino acid sequence identity with human ACE [17]. There are four other ACE-like genes in *D. melanogaster *that encode proteins which are predicted to be catalytically inactive as they lack critical residues involved in zinc binding or catalysis [18]. In the mosquito *Anopheles gambiae *there are 9 genes which code for proteins with similarity to mammalian ACE [19], while in *Caenorhabditis elegans *there is a single ACE-like gene which also encodes a protein that is predicted to be catalytically inactive [20]. Orthologues of ACE2 have been described recently in a range of non-mammalian vertebrates [21].

In this study we have identified and characterised a gene in the genomes of several mammalian species that encodes a novel, single domain ACE-like protein, that we have termed ACE3. In mouse, rat, cow and dog ACE3, the catalytic Glu is replaced by Gln (HQXXH) in the putative zinc binding motif, indicating that in these species ACE3 would lack catalytic activity as a zinc metalloprotease. In humans, we could find no evidence that the ACE3 gene is expressed and the presence of deletions and insertions in the sequence indicate that in humans ACE3 is a pseudogene.

## Results

### Predicted ACE3 sequences in mouse, rat, dog and cow genomes

Blast searches using human somatic ACE against the mouse genome and gene prediction programmes on the NCBI and Ensembl databases identified a potential homologous region on chromosome 11 (region E1) downstream of the ACE gene.

**Figure 1:**
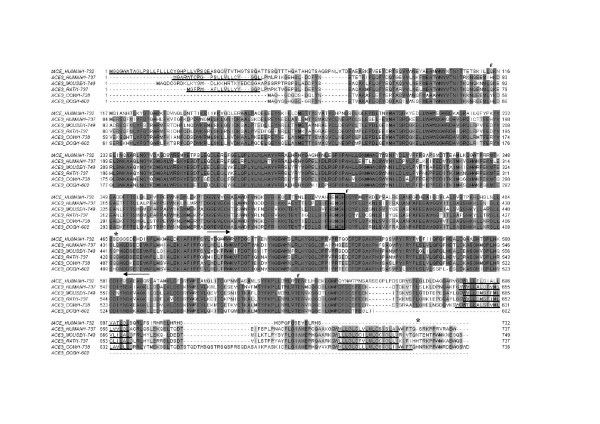
**Multiple sequence alignment of predicted ACE3 sequences from human, mouse, rat, cow and dog with human testicular ACE**. HEXXH motif indicated. Underlined are the predicted N-terminal signal sequences (human and rat ACE3) and C-terminal transmembrane regions (human, rat, mouse and cow ACE3), along with the known N-terminal signal sequence and C-terminal transmembrane region in testicular ACE. The human ACE3 sequence was restored by compensating for ^f^base deletion(s)/insertions and suppressing *stop codons. Positions of the forward and reverse RT-PCR primers are indicated by the arrows beneath the sequence. Accession numbers: Mouse XP_110936; Rat XP_573208; Dog XP_548034; Cow XP_600103.

The murine ACE3 gene consists of 2253 nucleotides which encodes a predicted protein of 750 amino acids (Fig. 1). Hydropathy analysis and transmembrane prediction revealed two hydrophobic regions at the C-terminus of the protein but no N-terminal signal peptide. The predicted amino acid sequence of murine ACE3 exhibits significant homology to existing members of the ACE family (Table 1), with the highest percentage identity (57%) to the C-terminal domain of murine somatic ACE. In murine ACE3, the HEMGH zinc binding motif characteristic of other members of the ACE family was HQMGH (Fig. 1). Predicted ACE3 sequences were identified also in the rat, dog and cow genomes (Fig. 1). These sequences are more closely related amongst each other than to ACE sharing 81%, 65% and 58% sequence identity with murine ACE3 in the case of rat, cow and dog, respectively. They all contain the HQMGH motif characteristic of mouse ACE3, however, neither the cow nor dog ACE3 predicted sequences contain an N-terminal signal peptide, although the rat ACE3 predicted sequence does. Both the rat and cow ACE3 sequences contain a predicted C-terminal transmembrane region, whereas the dog ACE3 lacks such a region.

**Table 1:** Percentage identity of the murine ACE family.

	**N-ACE**	**C-ACE**	**ACE2**	**ACE3**
N-ACE	100	52	43	46
C-ACE		100	42	57
ACE2			100	39
ACE3				100

The catalytic peptidase M2 domains [44] of murine ACE (N- and C-domain), ACE2 and ACE3 were aligned with each other using needle [37] and the percentage identity deduced.

### Genomic sequence analysis of the murine ACE3 gene

**Figure 2:**
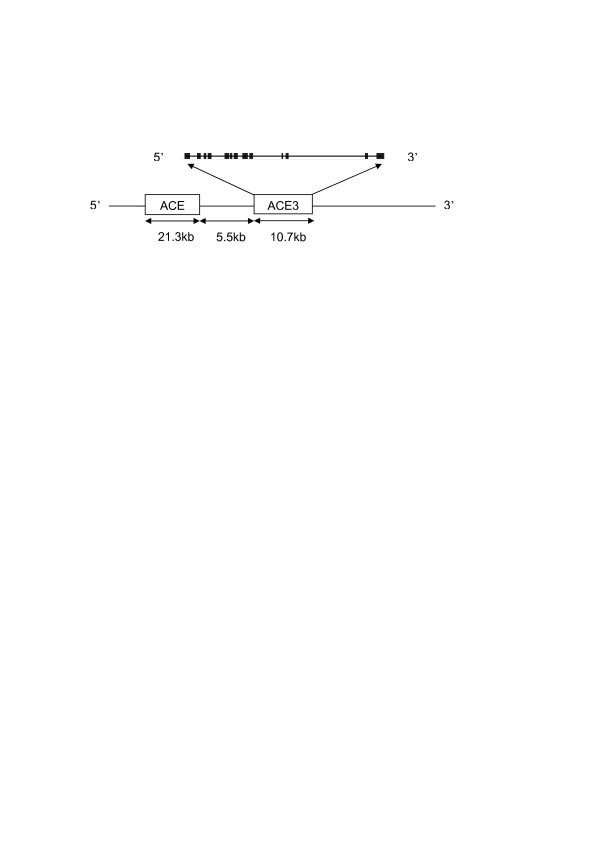
**Genomic mapping of mouse ACE3 to chromosome 11 region E1**. The exons encoding mouse ACE3 were mapped to the genomic sequence based on NCBI predictions for that gene.

The predicted murine ACE3 protein sequence was mapped onto the genomic DNA and the intron-exon boundaries determined. The murine ACE3 gene contains 13 exons, interspersed with 12 introns, spans 10.7 kb and is located 5.5 kb downstream of the ACE gene (Fig. 2). In the ACE3 gene, all the intron-exon junction sequences follow the GT/GA rule (Table 2). Exon 8 contains the HQMGH motif. The predicted ACE3 genes in rat, dog and cow are also located on the same chromosome as ACE, always downstream and in close proximity. A TblastN search of ACE3 against the annotated mouse genome at the NCBI only detected significant homology with ACE (e-value: 6 × 10^-79^) and ACE2 (e-value: 6 × 10^-10^). No other hit exists with an e-value of less than 0.01. This indicates that there is no further closely related homologue of ACE3, other than ACE and ACE2, in the mouse genome.

**Table 2:** Exon/intron boundary prediction for mouse ACE3.

**Exon no**.	**Exon size (bp)**	**cDNA position**	**5' Splice donor**	**Intron size (bp)**	**3' Splice acceptor**	**Codon phase**
1	264	1–264	catgGTGTGGT	385	CAGctgc	0
2	159	265–423	agagGTGACAG	261	CAGtaca	0
3	88	424–511	gcctGGTGAGCA	118	CAGACctcc	1
4	144	512–655	taatGGTAAGTG	740	TAGGAtaca	1
5	192	656–847	cctgGGTAAGGG	81	CAGGGaaca	1
6	98	848–945	ccagGTCAGTG	111	CAGcact	0
7	173	946–1118	cttcAGGTGTCTA	328	TAGGgtaa	2
8	224	1119–1342	ttcaGGTGATGG	152	CAGAGgagg	1
9	145	1343–1487	cttcAGGTGGGGA	1543	CAGGttga	2
10	99	1488–1586	tatcCGGTAAAGG	173	CAGAtact	2
11	123	1587–1709	cttgGGGTGAGTG	4092	TAGGgatc	2
12	185	1710–1894	tgaaGGTTGGAC	476	CAGAAaaaa	1
13	359	1895–2253				

Lower case letters denote exon sequences; intron sequences are in upper case letters. The codon phase indicates the position of the intron in the codon triplet. O intron occurs between codons, I intron occurs after the first nucleotide, and II intron occurs after the second nucleotide in the codon.

### Expression of ACE3 in murine tissues

**Figure 3:**
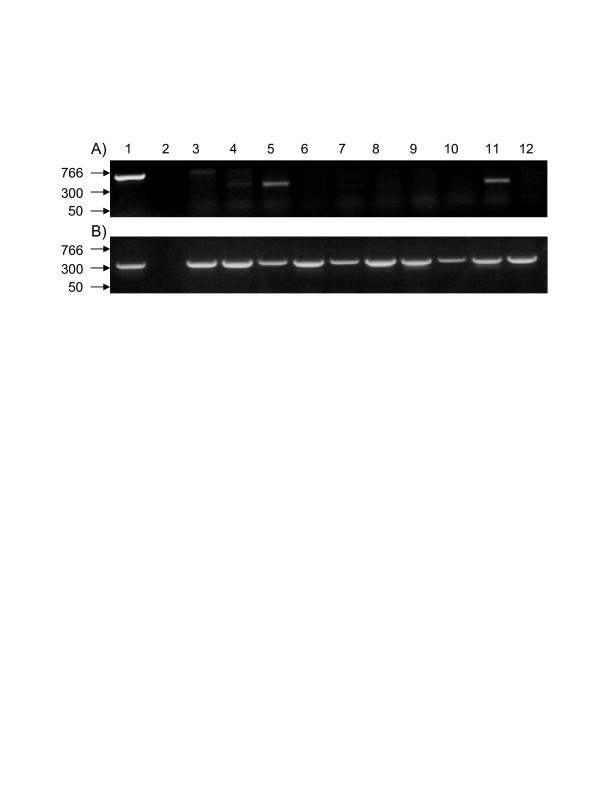
**RT-PCR analysis of ACE3 mRNA expression in various murine tissues**. Total RNA (1 μg) from the indicated tissues was subjected to one step RT-PCR as described in Materials and Methods followed by electrophoresis on a 1% agarose gel. (A) mouse ACE3; (B) actin. Lanes are as follows: 1, positive control (whole mouse RNA with primers for actin); 2, negative control (no RNA); 3, brain; 4, embryo; 5, heart; 6, kidney; 7, liver; 8, lung; 9, ovary; 10, spleen; 11, testis; 12, thymus.

The expression of the mRNA encoding ACE3 was examined in several murine tissues. RT-PCR using selective primers was performed on a panel of total RNA samples from different tissues (Fig. 3). A transcript encoding ACE3 was detected in testis, heart and embryo, but not in a range of other tissues, including, kidney, lung, and liver. Although a larger RT-PCR product was identified in the brain RNA sample, on sequencing this was found not to correspond to ACE3.

### Modelling of the substrate binding site of murine ACE3

**Figure 4:**
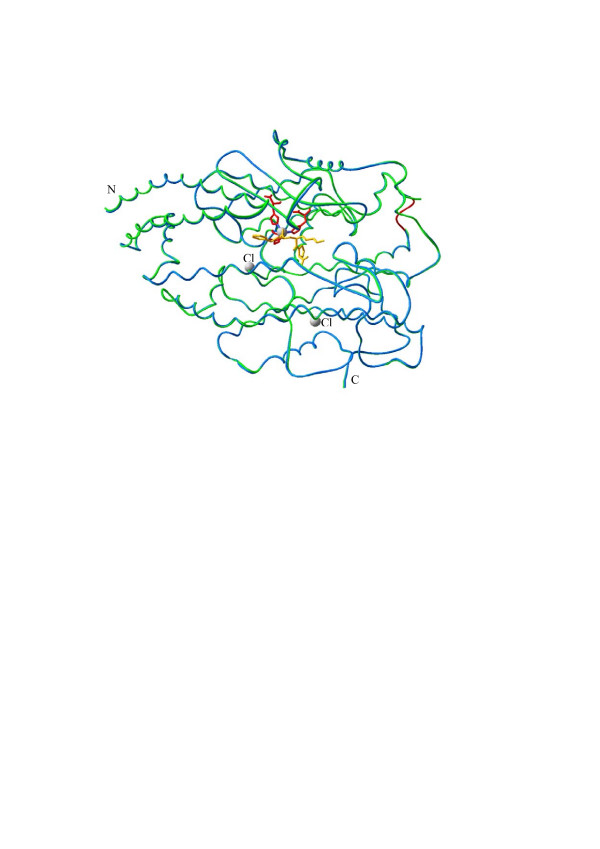
**Model of murine ACE3**. The murine ACE3 model (blue) is superimposed on the human testicular ACE structure (green) co-crystallised with lisinopril (yellow). The zinc ion (spacefill) is coordinated by the zinc binding residues (red). The chloride ions (Cl) of testicular ACE and the locations of the N- and C- termini are also shown. A loop missing in testicular ACE is modelled in red.

**Figure 5:**
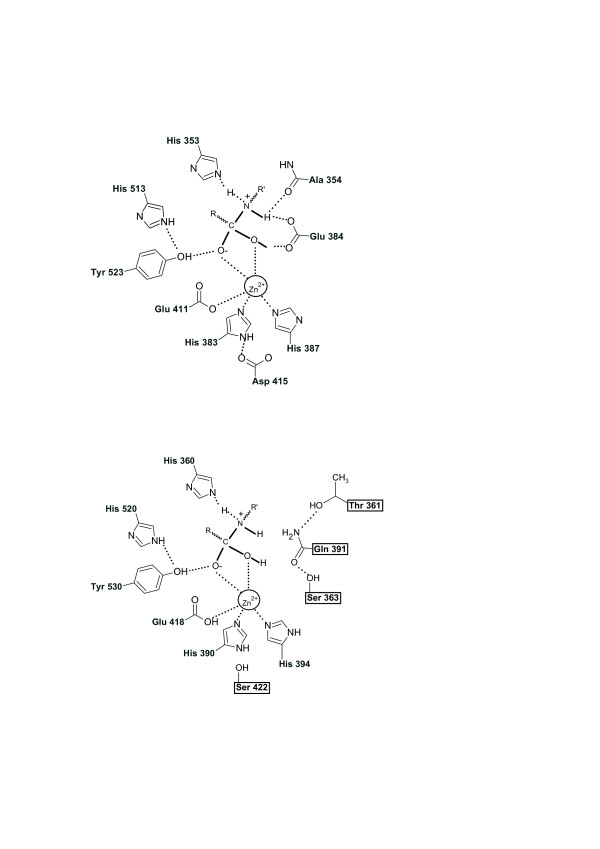
**Proposed catalytic mechanism in ACE (top) in contrast to predicted interactions in ACE3 (bottom)**. Four amino acid substitutions (in boxes) are rationalised to cause loss of activity in murine ACE3 (see text). Note: Gln391 can be flipped and interact either way with the hydroxyl groups of Thr361 and Ser363 as the hydroxyl group can act either as a hydrogen bond donor or as an acceptor.

The predicted murine ACE3 sequence was modelled on the C-terminal domain of human somatic ACE [5]. Overall, both the fold and active site in murine ACE3 are highly conserved as compared to human ACE (Fig. 4). However, several substitutions occur in the ACE3 predicted active site, some of which are unique to ACE3 family members (such as Glu384 to Gln and Asp415 to Ser) (Fig. 5). The most significant substitution is unquestionably Glu384 to Gln that replaces the catalytic Glu in the HEXXH zinc binding motif. Comparison of residues surrounding the proposed transition state of ACE [22,23] show why this substitution could be so detrimental to the ability of ACE3 to act as a protease (Fig. 5). Ionized Glu384 is proposed to stabilise the positively charged amine intermediate and act as a proton acceptor/donor in the ACE enzyme mechanism. In addition to the Gln being a poor proton acceptor/donor in ACE3, its involvement is further compromised by both the amide side chain atoms being involved in hydrogen bonding to two neighbouring residues, substituted from Ala to Thr361 and Ser363, respectively. Another important substitution is Asp415 to Ser which affects zinc binding by interfering with the hydrogen bond network to His383, the first histidine residue in the HEXXH zinc binding motif in testicular ACE.

### Genomic sequence analysis of the human ACE3 gene

**Figure 6:**
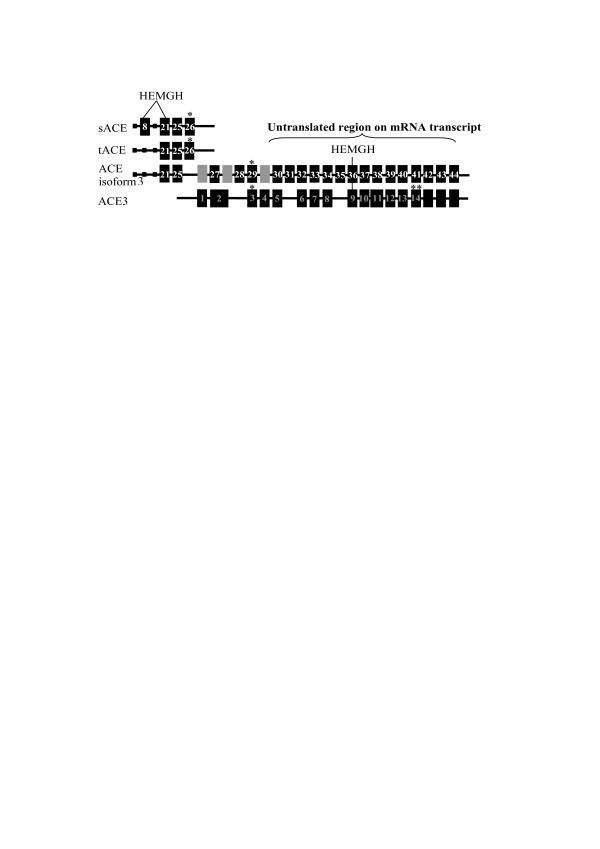
**Gene structure of the human ACE3 gene**. The restored ancestral ACE3 is compared to three known ACE transcripts (somatic ACE (sACE), testicular ACE (tACE) and ACE isoform 3). ACE3 can be partly reconstructed from the ACE isoform 3 transcript. For clarity, only relevant exons are shown. Exons (black), introns (grey). Exons numbered according to NCBI gene annotation (ACE) and by comparison to other ACE3 sequences (ACE3). Individual ACE3 exons are identical to isoform 3 except for exons 2 and 11 (both have extra base pairs). Exons 28, 31 and 35 are not part of ACE3 (non-ACE like exons). Exons 1, 4 and part of exon 2 are spliced out in the isoform 3 transcript. * indicates stop codons; ** restored ACE3 stop codon (adapted from NCBI GeneID:1636).

Blast searches using human somatic ACE against the human genome and gene prediction programmes on the Ensembl and NCBI databases identified a potential homologous region on chromosome 17 (region q23), 30 kb downstream of the start site of the ACE gene. This third potential human homologue of ACE was termed human ACE3. According to current annotation for the ACE gene (NCBI), a third ACE transcript exists. Alternative splicing at the 3' coding region results in a frameshift and a C-terminus that differs from the testicular ACE transcript in the last three exons. Interestingly, this transcript variant comprises another 15 exons in the 3' non-coding region, closely corresponding to the location of ACE3 (Fig. 6), suggesting that the ancestral ACE3 may be a pseudogene contained within the ACE gene.

The restored human ACE3 protein sequence (adjusted for several base deletions/insertions causing frame shifts and premature stop codons, see Fig. 1) shows strong conservation with ACE and other ACE3 sequences. It contains the typical HEMGH zinc binding motif present in other members of the ACE family and is 54% identical to the C-domain of human ACE and 71% identical to the mouse ACE3 catalytic domain, respectively. The ancestral ACE3 gene is predicted to contain at least 14 exons, interspersed with 13 introns and spans 13 kb (Fig. 6). All of the predicted intron-exon junction sequences follow the GT/GA rule (data not shown). Hydropathy analysis and transmembrane prediction revealed two hydrophobic regions at the C-terminus, in agreement with ACE3 from other species, and an N-terminal signal peptide.

The human ACE3 gene sequence, however, has several frame shifts and premature stop codons (see Fig. 1). For example, there is a one base deletion directly after the sequence encoding the HEMGH zinc binding motif that causes a frameshift and a premature stop codon. To confirm this deletion, genomic DNA was isolated from 3 different human cell lines (Eahy926, Hek293, SH-SY5Y) and PCR performed using primers flanking this region. In all three cell lines sequencing of the PCR product confirmed that there was a one base deletion directly after the codon for the second His of the zinc binding motif (data not shown).

RT-PCR was used in an attempt to identify potential mRNA transcripts of human ACE3. However, RT-PCR using various primer sets on an RNA panel that included total RNA from human brain, heart, kidney, liver, lung, testis, colon, small intestine, placenta and skeletal muscle failed to identify a transcript. The specificity of the primers was checked by using them to PCR genomic DNA extracted from Hek293 cells from which they amplified a band of the predicted size that corresponded to the sequence of ACE3 (data not shown). To date, blast searches of the EST databases has not identified any ESTs for human ACE3. Thus, point mutations that give rise to premature stop codons and the failure to identify RNA transcripts or ESTs all suggest that human ACE3 is likely a pseudogene.

## Discussion

In this study we have identified and characterised the gene encoding a third mammalian homologue of ACE, termed ACE3. The properties of the predicted ACE3 protein sequences in human, mouse, rat, dog and cow are summarised in Table 3. ACE3 in the mouse, rat, cow and dog all contain Gln rather than Glu within the HQMGH motif. The Glu residue is 100% conserved in all other ACE (M2) family members and the MA (HEXXH containing) clan of metalloproteases and is critically involved in catalysis [24]. When this Glu was mutated to Gln in either endopeptidase-24.15 [25] or ACE (Elliot, Duff, Jackson & Hooper, unpublished) enzyme activity was completely abolished. Another important substitution in murine ACE3 is Asp415 to Ser which affects zinc binding by interfering with the hydrogen bond network to the first histidine residue in the HEXXH zinc binding motif. This residue is conserved among vertebrate ACEs and in other metalloproteases, corresponding to Asp650 in neprilysin (M13 family) and Asp170 in thermolysin (M4 family). In both ACE and neprilysin, this Asp was shown to be critical for catalytic activity [26,27]. Due to these critical residues being mutated in ACE3, it is likely that in mouse, rat, cow and dog ACE3 will not be functional as a protease. Although the ACE3 sequences in mouse, rat and cow all possess C-terminal hydrophobic regions, only the rat ACE3 sequence is predicted to contain an N-terminal signal peptide which would direct the nascent protein into the lumen of the endoplasmic reticulum and then potentially to the cell surface as is the case for ACE and ACE2. ESTs for mouse, rat and cow ACE3 have been identified in the databases and RT-PCR of multiple tissues revealed a transcript for ACE3 in mouse heart and testis. In addition, a recent proteomic analysis of murine sperm regions that mediate sperm-egg interactions, reported the identification of a protein with the same gene locus (LOC217246) as ACE3 [28]. Thus, the available data suggest that ACE3 in mouse, rat and cow is expressed, but lacks protease activity.

**Table 3:** Comparison of the ACE3 sequences.

**Species**	**Length (amino acids)**	**Zinc-binding motif**	**Signal peptide**	**TM region**	**EST**
Human	737^§^	HEMGH			X
Mouse	750	HQMGH	X		
Rat	732	HQMGH			
Dog	602	HQMGH	X	X	X
Cow	738	HQMGH	X		

^§^refers to restored ancestral sequence as shown in Fig. 1.

Within another zinc metalloprotease family, the ADAM (a  disintegrin and metalloprotease) family, several members have subtle or more complete mutation of the HEXXH motif [29]. For example, in ADAM4 the Glu is mutated to Ala, while in ADAM29 it is His. Interestingly many of these catalytically inactive members of the ADAM family are present exclusively in the testis, where some have critical roles in fertilisation [29]. It remains to be determined whether ACE3 has a role to play in fertilisation, although the recent report of its presence in membrane vesicles released by the acrosome reaction [28] is suggestive of such a role. There is a further parallel between the ADAM family members and ACE3, in that a large number of the ADAMs present in the mouse genome are pseudogenes in humans [29]. In the present study we could find no evidence for human ACE3 to be expressed and the presence in the gene of multiple base insertions and deletions would, if expressed, generate a severely truncated protein lacking residues that are critical for zinc binding and/or catalysis in ACE and ACE2 [5,12,13]. This, and the localisation of ACE3 within the ACE gene, point to ACE3 being a pseudogene in humans.

Traditional, non-processed or duplicated pseudogenes characteristically contain introns as they are duplications of the genomic DNA, whereas processed or retrotransposed pseudogenes are duplicated from RNA and lack introns [30]. In addition, non-processed pseudogenes are usually adjacent to their original functional copies. As human ACE3 contains introns and is localised to the same region of the genome as ACE, it appears to be a non-processed pseudogene. Such non-processed pseudogenes are often silenced by point mutations, insertions or deletions, as seen in human ACE3. This 'pseudogenization' of genes in humans, as seen here with ACE3, results in the human degradome containing only 561 functional genes, while the mouse degradome is larger with 641 genes [31]. Interestingly, many of these additional mouse genes are involved in fertilisation and immunity.

While the ACE3 gene being located on the same chromosome just downstream of the ACE gene in all five genomes analysed may just reflect an evolutionary relationship, it is possible that this location alongside the ACE gene may reflect a potential functional role at the genomic level, as pseudogenes can regulate the expression of a related gene. For example, the makorin1 protein is evolutionarily conserved from nematodes to mammals and encodes an RNA binding protein [32]. Normally makorin1 is expressed throughout the animal but the disruption of the makorin1 pseudogene markedly reduced the expression of the makorin1 gene. This implies that for normal expression of makorin1, the presence of the RNA for the makorin1 pseudogene is required. However, it should be noted that a subsequent report failed to replicate this finding [33] and the technical problems associated with such studies are discussed more fully in [34]. Thus it is possible, but remains to be determined, that the ACE3 gene may regulate the expression of the ACE gene.

## Conclusion

Multiple ACE-like genes have been identified in a range of non-mammalian species and here we report the identification and characterisation of a third ACE-like gene in the genomes of several mammalian species. This gene encodes a novel, single domain ACE-like protein, ACE3. In the mouse, rat, cow and dog, where there is evidence that ACE3 is expressed, the catalytic Glu is replaced by Gln in the putative zinc binding motif, indicating that in these species ACE3 would lack catalytic activity as a zinc metalloprotease. In humans, although the predicted ACE3 protein would contain an intact HEXXH zinc binding motif, no evidence was found for the expression of the gene. This lack of expression, along with the presence of deletions and insertions in the sequence, indicate that in humans ACE3 is a pseudogene.

## Methods

### Bioinformatics Analysis

Blast searches for potential ACE3 sequences using human somatic ACE were performed against the mouse genome and gene prediction programmes on the NCBI [35] and Ensembl [36] databases. According to NCBI annotations, the alternate Celera genome assembly is composed of DNA from five different mouse strains: A/J, DBA/2J, 129X1/SvJ, 129S1/SvImJ and C57BL/6J, whereas the reference assembly is based on C57BL/6J. Sequences used for bioinformatics sequence analysis and homology modelling were obtained from the NCBI RefSeq database. Sequence analysis was performed using the Emboss software suite [37], Clustal W [38] and 3DCoffee [39]. Hydropathy analysis was performed as described [40]. Transmembrane and signal peptide regions were predicted using TMpred [41] and SignalP [42], respectively. Homology modelling of mouse ACE3 was undertaken with the SWISS-MODEL software [43] based on its alignment to the human testicular ACE template (PDB code 1o86).

### Reverse transcription-PCR

Human and murine (from Swiss Webster mice) total RNA was from Ambion (Europe) Ltd. (Huntingdon, UK). Genomic DNA was isolated from Eahy926, Hek293 and SH-SY5Y cells using TRIzol (GE Biosciences). Reverse transcription-PCR (RT-PCR) was performed with a one step Titanium RT-PCR kit (BD Biosciences, Oxford, UK) with 1 μg of RNA and the appropriate forward and reverse primers at 50 pmol/μl. For mouse ACE3 the primers were: forward 5'-GGGCGGGAAGTGGAGTGCCACA-3'; reverse 5'-GTTGACCTCCTCCTCTGAATCCTGG-3'. For human ACE3 the primers were: forward 5'-TCTGCCTGGAACTTCCCAGGACG-3'; reverse 5'-CCCATTTCGTGGAAAGATGGAGAGCG-3'. For mouse actin the primers were: forward 5'-GTGGGCCGCTCTAGGCACCAA-3'; reverse 5'-CTCTTTGATGTCACGCACGATTTC-3'. After incubation at 50°C for 1 h and initial denaturation for 5 min at 94°C, 30 cycles of denaturation at 94°C for 1 min, annealing at 55°C for 1 min and extension at 68°C for 1 min, followed by a final extension at 68°C for 2 min was performed.

## Abbreviations

ACE, angiotensin converting enzyme; ADAM; a disintegrin and metalloprotease; EST, expressed sequence tag; RT-PCR, reverse transcription-PCR.

## Authors' contributions

JLE, JF, AP, AJT and NMH identified the ACE3 gene. MR, JLE, JF and AP performed the genome sequence analyses. JLE and TJR performed the RT-PCR. MR and RMJ performed the molecular modelling. NMH, MR and RMJ drafted the manuscript. RMJ, AJT and NMH jointly planned the study and supervised experiments. All authors read and approved the final manuscript.
